# Requirement of CSR-1 isoforms and catalytic activity for *C. elegans* lifespan

**DOI:** 10.17912/micropub.biology.001676

**Published:** 2025-07-28

**Authors:** Brandon M. Waddell, Cheng-Wei Wu

**Affiliations:** 1 Veterinary Biomedical Sciences, University of Saskatchewan, Saskatoon, Saskatchewan, Canada

## Abstract

The *C. elegans* CSR-1 encodes an essential Argonaute protein that binds to 22 nucleotide small guide RNA to regulate germline gene expression. Recent characterization of the two CSR-1 isoforms (a and b) have demonstrated tissue-specific expression and functions. Here, we found that loss of function to the *csr-1 a* isoform has minimal effect on lifespan while a mutant with deletion to both *csr-1 a* and *csr-1 b* isoforms shows a significant decrease in lifespan. Furthermore, we found that single copy expression of a slicing inactive variant of *csr-1* fails to rescue the shortened lifespan. Overall, the data here provide new information on the requirement of isoform and domain-specific contribution of CSR-1 to *C. elegans* lifespan.

**Figure 1. Effect of csr-1 isoforms and catalytic activity on C. elegans lifespan:**
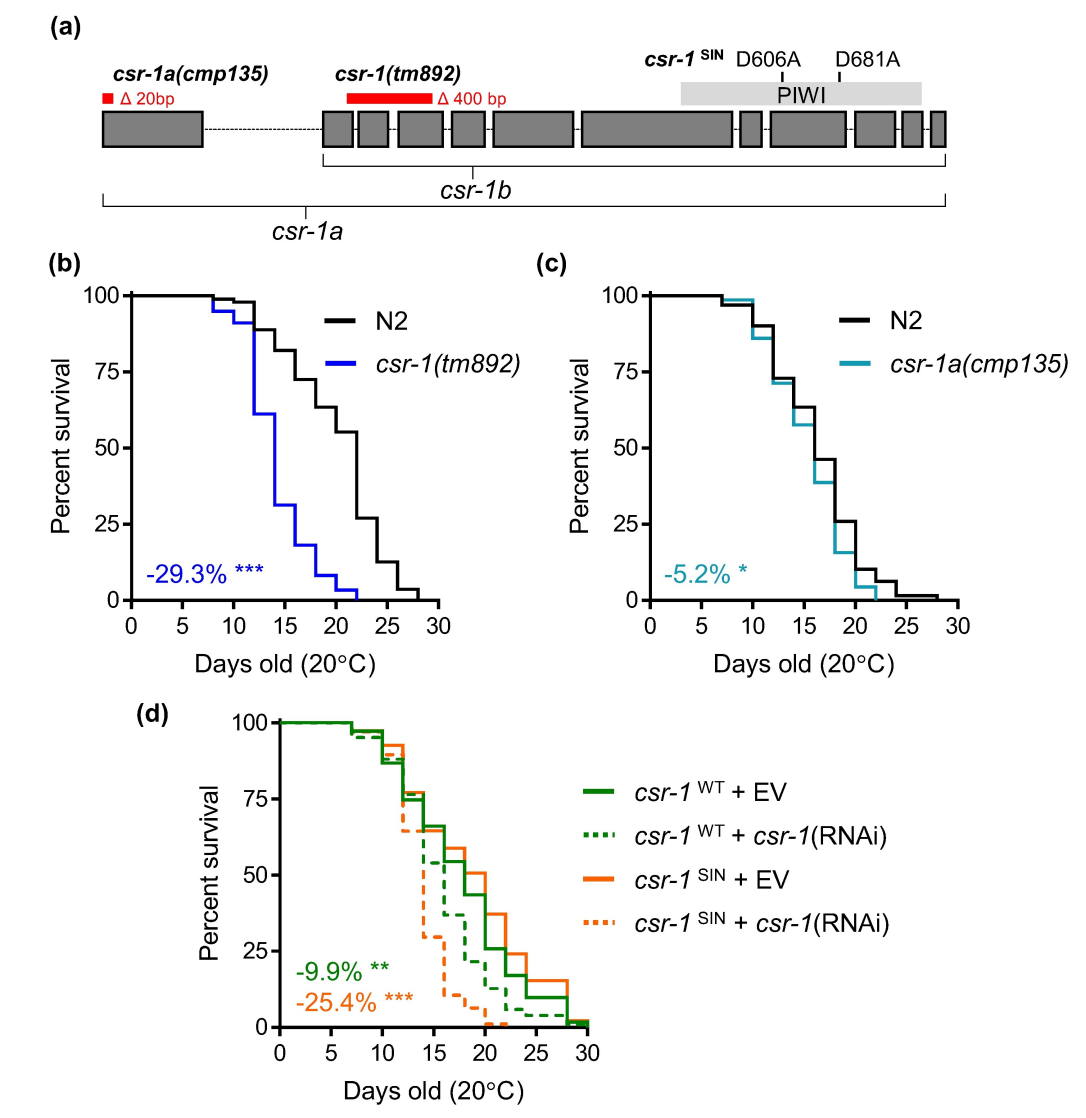
**(a)** Schematic of *csr-1* transcript illustrating the two isoforms and the PIWI domain. The location of nucleotide deletions in the mutants used is illustrated along with amino acids mutated within the PIWI region of the single copy *csr-1* ^SIN^ transcript. The *csr-1* sequence and domain information were retrieved from WormBase (Sternberg *et al.* 2024). Lifespan analysis of **(b)** *csr-1 ( tm892 )* and **(c)** *csr-1 a( cmp135 )* relative to N2 wildtype. **(d)** Lifespan analysis of *C. elegans* expressing a single copy of *csr-1* ^WT^ or *csr-1* ^SIN^ variant after feeding with empty vector (EV) or *csr-1* (RNAi). The *csr-1* (RNAi) targets a region within exon 6 of *csr-1 b* that is re-encoded in the single copy transcript to permit only knockdown of the endogenous *csr-1* transcript. The log-rank test was used to test for statistical significance of the lifespan assay, *P<0.05, ** P<0.01, and ***P<0.001. Three independent trials were performed for the lifespan assay with Trial 2 shown for all graphs. Statistics for all trials are presented in **Extended Data 1** .

## Description

The *C. elegans* CSR-1 ( C hromosome S egregation and R NAi deficient) encodes an essential Argonaute protein that binds to small guide RNA antisense to thousands of germline enriched transcripts to regulate gene expression (Claycomb et al *.* 2009; Cecere et al *.* 2014). Recently, we showed that *csr-1* is required for the 3' processing of small nuclear RNA (snRNA) transcripts in *C. elegans* , adding to the repertoire of cellular processes regulated by this Argonaute, which also positively processes histone mRNA 3' tail (Avgousti et al. 2012; Wedeles et al. 2013; Waddell and Wu 2024). A pair of studies have recently identified two isoforms of *csr-1* that exhibit distinct tissue and temporal-specific expression patterns (Nguyen and Phillips 2021; Charlesworth et al. 2021). In our study, we show that loss of *csr-1 a* does not influence snRNA processing, but a mutation that deletes both *csr-1 a* and *csr-1 b* isoforms led to an increase in misprocessed snRNA characterized by 3' cleavage failure and transcriptional read-through. Single copy rescue of catalytically inactive *csr-1 b* also fails to rescue snRNA misprocessing caused by *csr-1* depletion, implicating the requirement of the b isoform and its slicing activity for snRNA processing.

Loss of snRNA processing through knockdown or mutations to its principal regulator, the Integrator complex, shortens the lifespan of *C. elegans* and *Drosophila* (Tepe et al. 2023; Waddell and Wu 2024). To explore how *csr-1* influences lifespan, we performed lifespan analysis using *csr-1* isoform mutants and catalytically inactive variants. The *csr-1 ( tm892 )* mutant carries a 400 bp deletion generated by TMP/UV mutagenesis that renders both *csr-1 a* and *csr-1 b* null ( **Figure 1a** ). In the *csr-1 ( tm892 )* mutant, lifespan was drastically reduced by 29.3% compared to the N2 wildtype ( **Figure 1b** ). The *csr-1 a( cmp135 )* mutant contains a 20 bp deletion generated by CRSIPR edit that abolishes the start codon and the first seven amino acids of *csr-1 a* but does not affect *csr-1 b* ( **Figure 1a** ) (Nguyen and Phillips 2021). Loss of *csr-1 a* led to a small (5%) but statistically significant decrease in lifespan compared to the N2 wildtype ( **Figure 1d** ). A recent study has also shown that loss of *csr-1 a* has little to no effect on lifespan of *C. elegans* males (Rao et al. 2025). This data suggests that *csr-1 b* may play a more prominent role in lifespan regulation compared to *csr-1 a* , though we cannot rule out that the shortened lifespan of the *csr-1 ( tm892 )* mutant is caused by the loss of both isoforms.

Next, we investigated the requirement of the CSR-1 catalytic activity in lifespan regulation. We fed *C. elegans* strains expressing either a single copy of wildtype ( *csr-1 b* ^WT^ ) or s licing in active ( *csr-1 b* ^SIN^ ) variant of *csr-1* transcript empty vector (EV) or *csr-1* RNAi and measured lifespan. We used a *csr-1* RNAi clone that targets 420 bp within exon 6 of *csr-1 b* (or exon 7 of *csr-1 a* ), a region that is re-encoded in the single copy insertion variants to confer RNAi resistance. This strategy allows RNAi knockdown of the endogenous *csr-1* transcript to test the effect of the single copy variants that are unaffected by the RNAi (Gerson-Gurwitz et al *.* 2016; Waddell and Wu 2024). We found that *csr-1 b* ^WT^ showed a 10% decrease in lifespan when fed the *csr-1* RNAi while *csr-1* ^SIN^ showed a 25% decrease in lifespan compared to their respective EV fed control ( **Figure 1d** ). The moderate lifespan decreases in the *csr-1 b* ^WT^ strain suggest that single copy rescue is not sufficient to compensate for the depletion of endogenous *csr-1* caused by RNAi. However, the 25% lifespan decrease in the *csr-1 b* ^SIN^ strain suggests that the shortened lifespan is exacerbated when rescued with the slicing inactive variant of *csr-1 b* ( **Figure 1d** ) *.* This data suggests that the slicing activity of *csr-1 b* is required for a normal lifespan.

In conclusion, the analysis performed here provides new information on the isoform and catalytic specific requirements of *csr-1* on *C. elegans* lifespan. Given that loss of CSR-1 or its slicer activity leads to the upregulation of specific CSR-1 targets, resulting in embryonic lethality and sterility (Gerson-Gurwitz et al. 2016; Quarato et al. 2021; Singh et al. 2021), the observed effect of the *csr-1* phenotype on lifespan is likely due to pleiotropic factors, including developmental germline defects or altered stress responses.

## Methods

*C. elegans strain and lifespan assay*

*C. elegans* were cultured at 20°C using standard conditions described by described by (Brenner 1974). Strains used in this study are found in the Reagents table. For the lifespan assay, worms were synchronized using the hypochlorite treatment and grown on NGM agar plates containing 50 mg/mL carbenicillin and 100 mg/mLof isopropyl β-D-thiogalactopyranoside seeded with empty vector (L4440) or *csr-1* RNAi expressing HT115 (DE3) *E. coli* . Worms were manually transferred to new plates each day during the reproductive window for progeny separation and monitored every other day for death by gentle prodding with a sterilized platinum pick. Worms were considered dead if they did not respond to the prodding and were censored if they exhibited protruding gonad or vulva. N = 3 independent trials were performed for all lifespan studies, with the number of animals scored for each condition and statistics presented in Extended Data 1.

*csr-1 RNAi clone generation*

Genomic DNA from N2 wildtype worms were used to generate a 420 bp DNA fragment of *csr-1* within exon 6 flanked with *att* B cloning sites using primers listed in Reagents with Q5 High-Fidelity DNA polymerase. Gateway cloning was performed to first insert the template into pDONR221 followed by recombination into the pGC31 RNAi destination vector (Voutev and Hubbard 2008). The constructed *csr-1* RNAi clone was verified by Sanger sequencing and transformed into HT115 (DE3) *E. coli* .

*Statistical analyses*

The GraphPad Prism software (V8.4.3) was used to generate graphical data, with analysis of lifespan data performed using the log-rank test with OASIS2 ( https://sbi.postech.ac.kr/oasis2/ ) (Han et al *.* 2016).

## Reagents

**Table:** 

**Strain**	**Genotype**	**Description**	**Available from**
N2	Wildtype	Bristol wild isolate	CGC
OD923	* ltSi240 [ csr-1 p:: csr-1 (re-encoded) + Cbr-unc-119 (+)] II *	*csr-1b* ^WT^ , wildtype single copy insertion	CGC
OD925	* ltSi242 [ csr-1 p:: csr-1 (re-encoded; D606A, D681A: isoform b numbering) + Cbr-unc-119 (+)] II. *	*csr-1b* ^SIN^ , slicing inactive single copy insertion	CGC
WM182	*csr-1 ( tm892 ) IV/ nT1 [unc-?( n754 ) let-?] (IV;V) *	*csr-1a* and *csr-1b* null mutant	CGC
USC1258	*csr-1 a( cmp135 ) IV *	*csr-1a* null mutant	Dr. Carolyn Phillips

**Table:** 

**Primers**	**Sequence**	**Description**
*csr-1* RNAi *att* B1	GGGGACAAGTTTGTACAAAA AAGCAGGCTTCgtagcaggttatactcgaact	Forward primer to amplify *csr-1* exon 6 for RNAi. Uppercase indicate *attB1* sequence, lower case indicate *csr-1* primer binding sequence.
*csr-1* RNAi *att* B2	GGGGACCACTTTGTACAAGAAA GCTGGGTGccgcatttaatgttggcttt	Reverse primer to amplify *csr-1* exon 6 for RNAi. Uppercase indicate *attB2* sequence, lower case indicate *csr-1* primer binding sequence.

**Table:** 

**Plasmid**	**Genotype**	**Description**
pGC31	Gateway(R1-R2)-L4440	RNAi; Gateway destination vector

## Data Availability

Description: Extended Data 1. Resource Type: Dataset. DOI: https://doi.org/10.22002/d93b3-nkz22
